# A Preliminary Study of Aroma Composition and Impact Odorants of Cabernet Franc Wines under Different Terrain Conditions of the Loess Plateau Region (China)

**DOI:** 10.3390/molecules23051096

**Published:** 2018-05-05

**Authors:** Bao Jiang, Zhen-Wen Zhang

**Affiliations:** 1 Weinan Vocational & Technical College, Weinan 714026, Shaanxi, China; 2 College of Enology, Northwest A&F University, Yangling 712100, Shaanxi, China; happywine0618@sina.com

**Keywords:** wine, volatile compounds, terrain conditions, odor-activity values, SPME-GC/MS

## Abstract

Due to its appropriate climate characteristics, the Loess Plateau region is considered to be one of the biggest optimal regions for producing high-quality mountain wine in China. However, the complex landform conditions of vineyards are conducive to the formation of mountainous microclimates, which ultimately influence the wine quality. This study aimed to elucidate the influences of three terrain conditions of the Loess Plateau region on the aroma compounds of Cabernet Franc wines by using solid phase microextraction (SPME) with gas chromatography-mass spectrometry (GC-MS). A total of 40, 36 and 35 volatiles were identified and quantified from the flat, lower slope and higher slope vineyards, respectively. Esters were the largest group of volatiles, accounting for 54.6–56.6% of total volatiles, followed by alcohols. Wines from the slope lands had the higher levels of aroma compounds than that from flat land. According to their aroma-active values (OAVs), ethyl hexanoate, ethyl octanoate and isoamyl acetate were the most powerful compounds among the eight impact odorants, showing only quantitative but not qualitative differences between the three terrain wines. The shapes of the OAVs for three terrain wines were very similar.

## 1. Introduction

Aroma is one of the main factors contributing to the nature and quality of wine and sets the difference between a vast number of wines and wine styles produced throughout the world [1], therefore playing an important role in consumer preference [2]. Some of the aroma compounds come directly from the grapes, while others are formed during fermentation and ageing [3], so the existence of aroma compounds in wine is more complex than in grape berries. Describing the aroma of wines is not a simple task for researchers, because more than a thousand volatile compounds which are present at different concentrations have so far been identified in wine [4,5], such as alcohols, esters, fatty acids, aldehydes and ketones, etc., and these compounds present an extremely complex chemical pattern in both qualitative and quantitative terms, but their contribution to wine aroma does not depend only on the concentration, the perception threshold also plays an important role [6].

Solid phase microextraction, developed by Arthur and Pawliszyn [7] and Pawliszyn [8], has been considered as one of the most brilliant inventions in the field of sample preparation in recent years. Especially, headspace solid phase microextraction (HS-SPME) has been considered as a good choice for sample preparation in the aroma analysis [9]. Compared with conventional solvent extraction, HS-SPME is a fast, easy to use, inexpensive and solvent-free procedure for aroma and flavor studies [10]. HS-SPME coupled with GC or GC/MS has been widely applied to analyse and monitor the aroma of grapes and wines [11,12,13].

Since the aroma of young wines is at least partly the result of grape metabolism, many environmental factors, such as climate, soil, terrain (including exposure and altitude, etc.) have been acknowledged to greatly influence grape and wine quality [14]. Altitude can exert an important influence on grape maturation and wine composition that is strictly related to the local mesoclimate features, such as temperature, humidity, sunlight exposure, etc. Research on the aroma of Cabernet Sauvignon wine from Brazil indicated that wines from higher altitudes have a bell pepper aroma, while wines from lower altitudes are correlated with red fruit and jam aromas [15]; Reynolds et al. [16] have reported that in Canada, fruit and wine flavor components and sensory attributes overall in Gewürztraminer were responsive to vineyard site; in Italy, it has been reported that vineyard location has an influence on flavor compounds and wine quality by demonstrating that high monoterpene concentrations are associated with warm sites [17] and in the Yunnan Plateau of China, the number of volatile compounds in Cabernet Sauvignon wine increased with rising altitude, while concentrations of the total volatiles were decreased [18].

**Figure 1:**
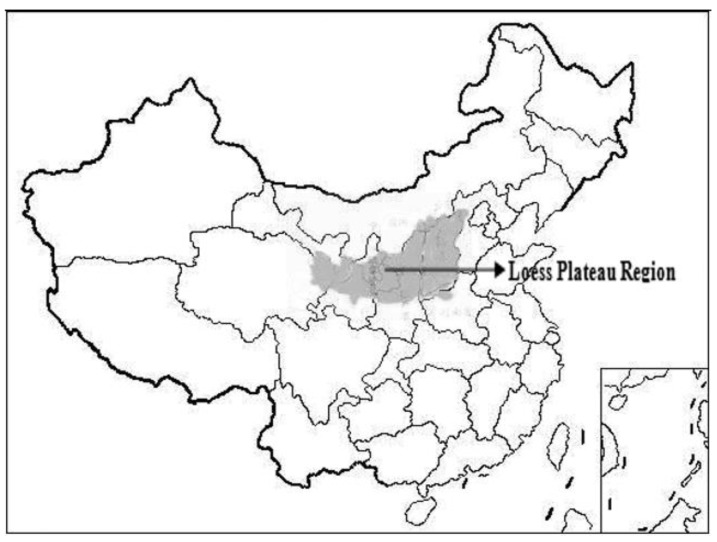
The distribution of the Loess Plateau region (China).

**Figure 2:**
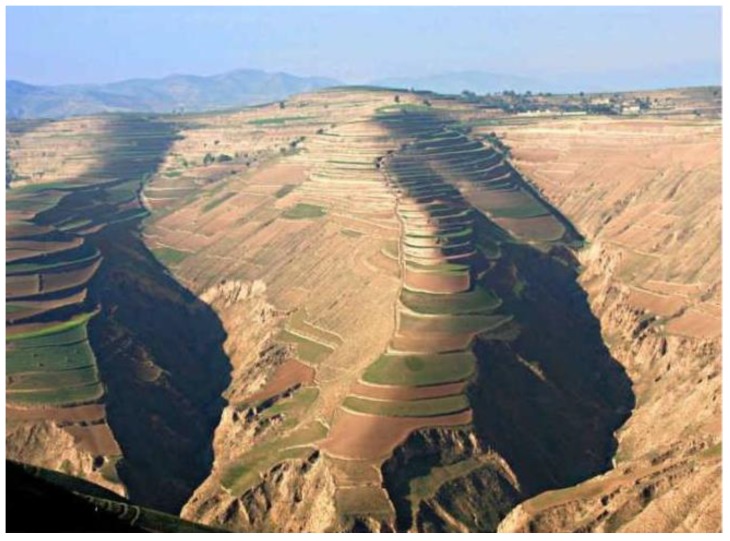
The photo of the typical topography and landform in the Loess Plateau region (China).

With the development of the Chinese wine industry, more and more wine-producing regions have been developed, including the Loess Plateau region of China which occupies about 600 thousand square kilometers (Figure 1). Rongzi Chateau of Xiangning County is located in the Loess Plateau region, where the different characteristics of the landform such as crisscross gulleys, different slopes, slope direction and altitude contribute together to form the local mountainous microclimate (Figure 2). The mean annual temperature and that of the coldest month (January) are 9.9 °C and −6 °C, respectively. Active accumulated temperature (≥ 10 °C) is more than 2998 °C with proper precipitation (annual rainfall around 50 cm). The climatic characteristics of semiarid climate, stronger sunshine, and a big temperature difference between daytime and night time create an especially healthy environment for vines in the Loess Plateau region. Cabernet Francs, which are well-known *Vitis vinifera* cultivars, is still among the most popular cultivars all over the world because of their strong adaptability and premium quality traits, but to date, the influences of terrain conditions of the Loess Plateau region on the aroma compounds of Cabernet Franc wines have not been documented.

In the present study, we analyzed the effects of vineyard terrains on the aroma compositions and impact odorants of Cabernet Franc wines, with volatiles being extracted by HS-SPME and detected by GC–MS. The objectives are to elucidate these wines’ characteristics using the OAVs of their monovarietal wines, which study could: (1) help winemakers optimize operational conditions (harvest parameters, juice preparation, fermentation techniques, use of yeasts, bacteria and enzymes, etc.) in order to emphasize one or more aromas in the final wines produced the Loess Plateau region; (2) provide some valuable information for producing high-quality mountain wines around the world.

## 2. Results and Discussion

### 2.1. Physicochemical Parameters

In order to monitor the effect of the different terrain conditions of the Loess Plateau Region on the Cabernet Franc wines, the physicochemical parameters of the Cabernet Franc wines from the flat land (F-Land) vineyard, low slope land (LS-Land) and high slope land (HS-Land) vineyards were determined (Table 1). 

Total sugar content of must was higher in F-Land wine than in the other two slope land wines, and total acidity content of the must showed no differences between the three terrain vineyards, but the sugar-acidity ratio from the three terrain berries were more than 20, so they all had a good ripeness. Total phenolics and pH of must displayed no obvious differences in the three terrain conditions. After fermentation, the wines from the three terrain conditions basically had similar physicochemical properties within an acceptable range, except for total phenolics contents [19]. Total phenolics contents of wines from F-Land vineyard was significant higher than from the slope land vineyards (*p* < 0.05), a discrepancy that perhaps affects the aroma characteristics of the corresponding wine.

### 2.2. Volatile Composition

A total of 44 compounds were identified and quantified in Cabernet Franc wines by GC-MS with HS-SPME (40, 36 and 35 different aroma compounds for F-Land, LS-Land and HS-Land, respectively), including 19 alcohols, 14 esters, five fatty acids, five aldehydes and ketones, and one phenol compound (Table 2). Moreover, alcohols and esters were the most represented compound classes in terms of the number and concentration of volatile compounds in the three terrain Cabernet Franc wines, and fatty acids, volatile phenols, aldehydes and ketones were detected as minor compounds. Many of these volatile compounds are common to most of the wines and are derived from the grape berries and yeast strains during the fermentation and the vinification process [20].

In this study, a wide concentration range of the total volatile compounds varying from 230.1 to 367.2 mg/L was quantified in the three terrain wines. The total amounts of aroma compounds detected from HS-Land wine was the highest, while the levels in wine from F-Land were the lowest. The results in this study were consistent with the results of a previous study which was carried out with the Cabernet Sauvignon (*Vitis vinifera* L. cultivar) wines from vineyards at different altitudes by Yue et al. [18]. It is well known that the aroma of wine predominately depends on many factors, including grape variety, environmental and management practices, yeast, winemaking techniques, ageing time, etc. [13,21]. Since in the present study, the management practices, ageing time, the yeast and the fermentation conditions used were the same for all treatments, differences in compound concentrations could be explained by the different altitude and its related climatic conditions in vineyard. To further illustrate the differences in the Cabernet Franc wines from the three different terrain conditions, a comparison of the subtotal of each aroma subclass among the three terrain wines was made.

Alcohols are produced by yeast during alcoholic fermentation [22], and usually have a strong and pungent smell, as well as taste. In our study, 18, 16 and 15 higher alcohols were identified in F-Land, LS-Land and HS-Land Cabernet Franc wines, respectively. Alcohols were the largest group in terms of the number of aroma compounds identified in three terrain wine studied, followed by esters, fatty acids, aldehydes and ketones. The subtotal concentration of alcohols in three terrain Cabernet Franc wines was from 94.6 to146.9 mg/L, which made up of 40.0–41.4% of the total aroma compounds detected. At concentrations below 300 mg/L, they contribute to the desirable complexity of wine, but when their concentrations exceed 400 mg/L, higher alcohols have a negative quality factor [23]. In the current study, the alcohols might have a positive contribution to the overall aroma of the three examined wines as their levels are below 300 mg/L. Compared with the F-Land wine, in addition, the Cabernet Franc wine from two slope land vineyards had higher content of alcohols, especially HS-Land condition wine (Table 2).

Among detected alcohols, isoamyl alcohol, 2-phenylethanol, 1-propanol and isobutyl alcohol were the dominant alcohols in all wine samples, and showed higher content in each terrain wine, which had concentrations of > 14 mg/L (except for isobutyl alcohol and 2-phenylethanol in wine from F-Land), in agreement with previous studies [13]. Furthermore, among the dominant alcohols, even though isoamyl alcohol and 2-phenylethanol had higher contents than the respective odor threshold, both had concentrations much lower than 400 mg/L, thus contributing in a positive way to wine aroma [24]. Isoamyl alcohol was the most abundant alcohol, accounting for 38.9–53.9% of the total alcohols in the three terrain wines, it contributes cheese sensory properties to wine aroma. Compared with the slope land wines, the alcohol profile of the F-Land wine was more diverse, containing 18 types of alcohols compared with only 15–16 in two slope land wines. 1-Octen-3-ol and 2-octanol were only present in the wine made from the flat land Cabernet Franc wine.

Acetate esters are the result of the reaction of acetyl-CoA with higher alcohols that are formed from degradation of amino acids or carbohydrates [25]. Ethyl acetate, isoamyl acetate, hexyl acetate, phenethyl acetate and heptyl acetate were the detected acetate esters. The analyzed acetic acid esters are considered as factors contributing to quality in young wines [26]. Although, their amount varied between three terrain wines, ethyl acetate and isoamyl acetate were the major esters found in the aroma components of the different terrain wines in terms of their concentrations, and their total concentrations were 66.8 mg/L and 117.5 mg/L (average value of two slope land wines), respectively in the flat and slope land wines, which are perceived as having a fruity and banana flavor. The concentration of ethyl acetate in the each slope land wine was nearly 2.0 times that in the flat land wine, which implied the slope land wines could have an enhanced fruity aroma, therefore, ethyl acetate could be a potential impact odorant of wines containing this chemical.

Another group of volatile esters in wine are the ethyl esters of fatty acids that are produced enzymatically during yeast fermentation and from ethanolysis of acetyl-CoA that are formed during fatty acids synthesis or degradation. Their concentration is dependent on several main factors: yeast strain, fermentation temperature, aeration degree and sugar content [25]. A total of six ethyl esters were detected and quantified in three wine samples. The esters of this group make a positive contribution to the general quality of wine. Most of them have the typical fruity aroma of young wines [27]. Among these ethyl esters, the most abundant compounds were ethyl hexanoate, ethyl octanoate and ethyl lactate, which all exhibited higher concentration in slope land wines than those of the F-Land wine. As compared with the upper three ethyl ester compounds, the contents of ethyl dodecanoate was lower in three terrain wines, especially in F-Land wine, but the ethyl dodecanoate can cause more abundant and complex wine aromas [28]. Furthermore, this study and previous research shows that the composition and concentration of the wine aroma could be regulated by the position of the vineyard [29].

The production of fatty acids has been reported to be dependent on the composition of the must and fermentation conditions [30]. Five fatty acids were detected in all wine samples, and the content of each fatty acid detected from the F-Land wine showed the lowest than those from two slope land vineyards. Octanoic acid, hexanoic acid and decanoic acid were the major fatty acids found, however, the contents of isobutyric acid and heptanoic acid were very low in all wine samples (and existed in at least one of the wines studied), especially for heptanoic acid, it is well known that both of them are not associated with wine quality but play an important role in the complexity of the aroma [31]. Specifically, they are important for the aromatic equilibrium in wines because they are opposed to the hydrolysis of the corresponding esters [32]. Appropriate content of fatty acids was necessary for higher contents of aroma esters in wines. These C_6_ to C_10_ fatty acids at concentrations of 4 to 10 mg/L impart mild and pleasant aromas to wine; however, at levels beyond 20 mg/L, their impact on wine becomes negative [31]. The C_6_ to C_10_ fatty acids might have a positive impact on the aroma of the three wines examined in the current study since their levels were all far below 10 mg/L.

The composition and concentration of aldehydes and ketones varied among the different terrain wines. Acetoin, benzaldehyde, benzylethylaldehyde, nonanal and geranylactone were found in these wine samples. The concentration of aldehydes and ketones class from the LS-Land Cabernet Franc wine was higher than in other terrain wines.

The identification of volatile phenols in wine (phenol) can have an influence on the aroma of the wine. Those yeast strains that are naturally present on the grapes and in the winery such as *Brettanomyces* yeasts can also contribute to the production of volatile phenols [33]. In addition to the metabolic activity of yeasts, other factors such as oak maturation can also increase the amounts of volatile phenols in wine [34]. In present study, the phenol was found in the all wine samples, but it was only present at very low amount.

### 2.3. Odor-activity Values (OAVs)

Though dozens of volatiles were detected in each terrain wine, but not all of the components have the same impact on the overall aroma character of this wine. Of all the compounds detected, only those displaying OAVs greater than 1 were deemed to contribute to wine aroma [5]. By the OAVs we can estimate the contribution of specific compound to the overall wine aroma.

Table 3 shows total 13 OAVs for compounds that exceeded their thresholds in the three terrain Cabernet Franc wines, and thereby they all possibly contributed to the wine aroma. Three of these were the most powerful compounds in three terrain wines: ethyl hexanoate, ethyl octanoate and isoamyl acetate, especially ethyl hexanoate, although aroma synergy and suppression exist, all of them are byproducts of yeast metabolism, they were responsible for the fruity, floral and anise sensory properties of young wine. Aside from isoamyl acetate, the OAVs of both ethyl hexanoate and ethyl octanoate from the slope lands were the higher than that in the flat wine; they could exert a strong influence on wine aroma. Such differences might be attributed in part to the specific “terrain” factor. The vineyards are located in slope lands (LS-Land and HS-Land) with average altitude of 1352 m above sea level. It provides with a lower temperature, a wide swing in diurnal temperature differences distinguished by lower night-time temperature, high UV radiation and light intensity. These specific characteristics might stimulate the ethyl octanoate metabolism. The results in our study partially agreed with previous report [13] indicating that these were also the upper three most powerful odorants according to the OAVs of aroma compounds in the Cabernet Franc wines from Huailai County of China, but ethyl octanoate was the first predominant odorant among of them, which accounted for 71.8% of the global aroma of Cabernet Franc wine rather than was ethyl hexanoate as present study. Five components had OAVs higher than or very close to unity in three terrain wines: ethyl acetate, octanoic acid, ethyl decanoate, 3-(methylthio)-1-propanol and isoamyl alcohol. Among them, 3-(methylthio)-1-propanol and octanoic acid had some bad effect on the overall wine aroma, this is because both compounds share boiled potato, rubber and rancid, harsh sensory properties; but the other three give a pleasant character which are described as having fruity and cheese odor. Finally, the other compounds quantified in Table 3 can be considered as occasional odorant, they can reach OAVs higher than their corresponding odor threshold in some wine samples, but lower in other wines. Although some aroma compounds could be present at sub-threshold concentrations (i.e., OAVs < 1), their potential contribution to wine aroma should not be excluded, because they can enhance some existing notes by synergy with other compounds [44].

Taking into consideration the OAV of each individual compound, the aroma profiles for the Cabernet Franc wines from the three different terrain vineyards were analyzed. For the three different terrain wines, differences existed in the shape of the OAVs of each wine, especially ethyl butanoate. But the overall shapes for all the wines were very similar, showing only quantitative but not qualitative differences. In addition to variety factor, this might be related to the same “terroir” characteristics between these vineyards, resulting in similar aroma profiles.

## 3. Materials and Methods

### 3.1. Chemicals

All standards were purchased from Fluka (Buchs, Switzerland) and Aldrich (Milwaukee, WI, USA). Purity of all standards was above 99%. 4-Methyl-2-pentanol was employed as the internal standard. Model solutions were prepared using the methods reported by Howard et al. [45]. For quantification, 8-point calibration curves for each compound were prepared using the method described by Ferreira et al. [46], which was also used as a reference to determine the concentration range of standard solutions. 

### 3.2. Sample Collection and Vinification

The present study was conducted for the 2016 vintage using *Vitis vinifera* cv. Cabernet Franc vines grafted onto SO4 rootstock, grown on a commercial chateau. Vines were aged 5 years, Dulong-trained, with a vine spacing of 2.5 × 1.0 m. The vines were watered by drip irrigation system and were managed in accordance with the standard agronomic practices in the area. Soil was managed with cover grass. The original “Cabernet Franc” grape berries (200 kg per sample, totally 600 kg grape samples) were collected from three different terrain conditions of the Loess Plateau region, including F-Land vineyard, LS-Land and HS-Land vineyards (Table 4).

All grape berries were harvested manually at optimum technological maturity for these vineyards in September, 2016, as judged by the ratio of sugar and acid content. Pre-fermentation treatments and winemaking were performed according to Li et al. [47] Briefly, grapes were crushed on an experimental destemmer-crusher and then transferred to stainless-steel containers. Thirty L of each treatment wine were produced in three replicates. Fifty mg/L of SO_2_ and 30 mg/L of pectinase (Lallzyme Ex) were added to the musts and the contents were mixed by hand. After maceration of the musts for 24 h, 200 mg/L of dried active yeast (*Saccharomyces cerevisiae* strain, Lallemand, Danstar Ferment AG, Switzerland) was added to the musts, according to commercial specifications. Alcoholic fermentation was carried out at 20 to 25 °C to dryness (reducing sugar < 4 g/L) which took place over a 6–8 days period and density controls were maintained during this period. At the end of alcoholic fermentation the wines were separated from pomace, and then added 50 mg/L of SO_2_. After fermentation, the wine samples were bottled and stored at 10–15 °C prior to analysis. All the samples were five months old at the time of analysis. Total sugar, total acidity, pH, residual sugar and ethanol were analyzed [48], total phenolics was determined according to the Folin-Ciocalteu colorimetric method [49].

### 3.3. HS-SPME Procedure

Volatile compounds of all wine samples were extracted by HS-SPME and analyzed using gas chromatography/mass spectrometry as described by Zhang et al. [13]. Five milliliters of wine sample and 1 g NaCl were placed in a 15 mL sample vial. The vial was tightly capped with a PTFE-silicon septum and heated at 40 °C for 30 min on a heating platform agitation at 400 rpm. The SPME (50/30-μm DVB/Carboxen/PDMS, Supelco, Bellefonte, PA, USA), preconditioned according to manufacturer’s instruction, was then inserted into the headspace, where extraction was allowed to occur for 30 min with continued heating and agitation by a magnetic stirrer. The fiber was subsequently desorbed in the GC injector at 250 °C for 25 min.

### 3.4. GC–MS Analysis

The GC-MS system used was an Agilent 6890 GC equipped with an Agilent 5975 mass spectrometer (Agilent Technologies Santa Clara, CA, USA). The column used was a 60 × 0.25 mm HP-INNOWAX capillary with 0.25 μm film thickness (J & W Scientific, Folsom, CA, USA). The carrier gas was helium at a flow rate of 1 mL/min. Samples were injected by placing the SPME fiber at the GC inlet for 25 min with the splitless mode. The oven’s starting temperature was 50 °C, which was held for 1 min, then raised to 220 °C at a rate of 3 °C/min and held at 220 °C for 5 min, transfer-line temperature was 105 °C. The mass spectrometry in the electron impact mode (MS/EI) at 70 eV was recorded in the range m/z 20 to 450 u.m.a. The mass spectrophotometer was operated in the full scan and the selective ion mode (SIM) under autotune conditions at the same time. The area of each peak was determined by Chem. Station software (Agilent Technologies). Analyses were carried out in triplicate. Retention indices were calculated after analyzing C8-C24 *n*-alkane series under the same chromatographic conditions. Identifications were based on MS matching in the standard NIST05 library, retention indices of reference standard in authors’ laboratories and a comparison of retention indices reported in the literature. Retention indices were listed in Table 2.

### 3.5. Odor-activity Values (OAVs)

The specific contribution of each volatile compound to the overall wine aroma was determined by calculating the odor-activity value (OAV) as the ratio of the concentration of each compound to its detection threshold concentration [50].

### 3.6. Statistical Analysis

Data were reported as the mean ± SD. Statistical analyses were performed using the SPSS 16.0 for Windows (SPSS Inc, Chicago, IL, USA) with three replicates of the same sample. Significant differences between wines from different terrains were determined by Turkey’s test (*p* < 0.05).

## 4. Conclusions

This is the first in-depth study on effect of terrains on the volatiles of Cabernet Franc wines grown in the Loess Plateau region of China. In this study a total of 40, 36 and 35 volatile compounds were identified and quantified in F-Land, LS-Land and HS-Land Cabernet Franc wines, respectively. Esters were the largest group of volatile compounds, representing 54.6–56.6% of the total volatiles, followed by alcohols. Differences were also observed in the volatile compounds studied as a function of the terrain. The highest content of volatile compounds was found in the Cabernet Franc wines from the slope land vineyards compared with the flat land vineyard. Eight volatile compounds were always present in the three terrain wines with OAVs of more than 1—ethyl hexanoate, ethyl octanoate, isoamyl acetate, ethyl acetate, octanoic acid, ethyl decanoate, 3-(methylthio)-1-propanol and isoamyl alcohol—especially ethyl hexanoate, ethyl octanoate and isoamyl acetate, which were considered to be the most powerful odorants in wines, responsible for the fruity, floral and anise sensory properties of young Cabernet Franc wines. Furthermore, wine from the flat land seems to have more intense fruity aroma (banana) with less pineapple and pear attributes. According to the results of this study, further study is needed to evaluate the effect of ageing time on the volatile composition patterns of upper mountain wine samples.

## Figures and Tables

**Table 1:** General composition of the musts and wines of Cabernet Franc from the different terrains.

Analytical Parameters	F-Land		LS-Land		HS-Land	
	Must	Wine	Must	Wine	Must	Wine
Total sugar (g/L)	210.7 ± 2.2 ^A^	NA	198.5 ± 1.8 ^A^	NA	197.4 ± 0.9 ^A^	NA
Total acidity ^1^ (g/L)	7.5 ± 0.5 ^A^	9.6 ± 0.0 ^a^	7.2 ± 0.3 ^A^	9.3 ± 0.2 ^a^	6.9 ± 0.1 ^A^	9.5 ± 0.2 ^a^
pH	3.2 ± 0.1 ^A^	3.2 ± 0.1 ^a^	3.3 ± 0.1 ^A^	3.2 ± 0.2 ^a^	3.1 ± 0.1 ^A^	3.1 ± 0.2 ^a^
Total phenolics ^2^ (mg/kg or mg/L)	2306.2 ± 152.4 ^A^	889.7 ± 56.8 ^a^	2403.1 ± 96.0 ^A^	707.4 ± 20.5 ^b^	2306.1 ± 102.3 ^A^	660.9 ± 33.6 ^b^
Residual sugar (g/L)	NA	2.5 ± 0.1 ^a^	NA	2.2 ± 0.0 ^a^	NA	1.2 ± 0.2 ^b^
Ethanol (%, v/v)	NA	11.8 ± 0.2 ^a^	NA	11.6 ± 0.1 ^a^	NA	12.1 ± 0.1 ^a^

Each data in the table was mean values ± standard deviation of triplicate samples. Different capital letters within a row for must indicated significant differences among three terrain wines by Tukey’s test (*p* < 0.05). Different lower-case letters within a row for wine indicated significant differences among three terrain wines by Tukey’s test (*p* < 0.05). F-Land, LS-Land and HS-Land represented flat land, low slope land and high slope land conditions of experimental vineyards, respectively. NA, not apply. ^1^ Total acidity expressed as grams of tartaric acid equivalents per liter. ^2^ Total phenolics from grape must and wines expressed as milligrams of gallic acid equivalents per kilogram and milligrams of gallic acid equivalents per liter, respectively.

**Table 2:** GC-MS analytical results of aroma components in Cabernet Franc wines from the different terrains.

Compounds	RI	Threshold (mg/L)	Sensory properties	Concentration (μg/L)		
				F-Land	LS-Land	HS-Land
Alcohols						
1-Propanol	1057	306 [35]	Fresh, alcohol	16659.0 ± 880.5 ^c^	30857.9 ± 1609.7 ^a^	21572.9 ± 503.0 ^b^
Isobutyl alcohol	1111	40 [5]	Fusel, alcohol	10060.8 ± 72.4 ^c^	14970.7 ± 144.7 ^b^	19902.2 ± 112.6 ^a^
1-Butanol	1149	150 [35]	Medicinal, alcohol	1332.0 ± 140.3 ^b^	1487.7 ± 136.7 ^a^	1207.7 ± 18.5 ^c^
Isoamyl alcohol	1209	30 [5]	Cheese	51052.5 ± 36.8 ^c^	55280.9 ± 1131.0 ^b^	67458.6 ± 759.6 ^a^
1-Pentanol	1268	80 [36]	Fruity, balsamic	6.0 ± 0.5	nd	13.4 ± 1.3
4-Methyl-1-pentanol	1309	50 [37]	NA	nd	132.7 ± 4.8	nd
1-Hexanol	1348	8 [5]	Green, grass	1304.2 ± 13.2 ^c^	2851.6 ± 49.3 ^a^	1559.3 ± 160.9 ^b^
(*E*)-3-hexen-1-ol	1354	4 × 10^−1^ [5]	Green, floral	16.5 ± 0.6 ^c^	55.1 ± 2.3 ^a^	45.3 ± 5.5 ^b^
(*Z*)-3-hexen-1-ol	1378	4 × 10^−1^ [5]	Green	35.4 ± 2.5 ^c^	580.2 ± 10.1 ^a^	107.1 ± 7.8 ^b^
2-Octanol	1417	1.3 × 10^−1^ [36]	NA	tr	nd	nd
1-Octen-3-ol	1445	NA	NA	38.2 ± 2.4	nd	nd
1-Heptanol	1448	1 [36]	Grape, sweet	71.9 ± 0.9 ^c^	191.9 ± 9.6 ^a^	88.9 ± 5.0 ^b^
*levo*-2,3-Butanediol	1542	120 [38]	Butter, creamy	183.2 ± 10.4 ^b^	805.8 ± 3.5 ^a^	791.2 ± 45.4 ^a^
1-Octanol	1554	1.3 × 10^−1^ [36]	Intense citrus, roses	17.6 ± 0.7 ^b^	39.8 ± 1.2 ^a^	3.3 ± 0.3 ^c^
3-(Methylthio)-1-propanol	1726	5 × 10^−1^ [5]	Boiled potato, rubber	2041.4 ± 141.3 ^b^	2796.6 ± 43.2 ^a^	2900.4 ± 66.9 ^a^
1-Decanol	1781	4 × 10^−1^ [5]	Orange flowery, special fatty	10.9 ± 0.7	12.8 ± 0.3	nd
Benzyl alcohol	1894	200 [39]	Citrusy, sweet	352.3 ± 2.7 ^b^	604.8 ± 4.5 ^a^	622.7 ± 14.6 ^a^
2-Phenylethanol	1928	10 [5]	Flowery, pollen, perfumed	11434.8 ± 608.3 ^b^	31433.8 ± 1228.5 ^a^	30600.5 ± 50.7 ^a^
Citronellol	1767	1 × 10^−1^ [5]	Green lemon	2.6 ± 0.1 ^c^	14.0 ± 0.3 ^a^	5.1 ± 0.1 ^b^
Subtotal (μg/L)				94619.3	142116.3	146878.6
Proportion (%)				41.1	41.4	40.0
Esters						
Ethyl acetate	877	7.5 [5]	Fruity, sweet	64571.6 ± 298.4 ^c^	107130.0 ± 889.6 ^b^	123756.8 ± 928.3 ^a^
Ehyl butanoate	1032	2 × 10^−2^ [5]	Sour fruit, fruity	nd	nd	265.8±23.0
Ethyl hexanoate	1232	5 × 10^−3^ [5]	Fruity, anise	51090.6 ± 60.4 ^b^	63860.7 ± 364.7 ^a^	64627.6 ± 501.9 ^a^
Phenethyl acetate	1830	2.5 × 10^−1^ [5]	Pleasant, floral	8.1 ± 0.3 ^b^	31.8 ± 1.7 ^a^	36.9 ± 4.4 ^a^
Isoamyl acetate	1122	3 × 10^−2^ [5]	Banana	2238.0 ± 113.8 ^a^	1569.5 ± 29.3 ^b^	2051.5 ± 59.5 ^a^
Hexyl acetate	1287	6.7 × 10^−1^ [35]	Pleasant fruity, pear	178.1 ± 3.1 ^a^	125.1 ± 6.6 ^c^	154.9 ± 10.7 ^b^
Ethyl lactate	1363	14 [40]	Lactic, raspberry	4203.7 ± 124.4 ^c^	4477.4 ± 20.9 ^b^	5646.6 ± 100.1 ^a^
Heptyl acetate	1051	1.4 [36]	Almond, pear	1.1 ± 0.2 ^b^	2.1 ± 0.3 ^a^	1.5 ± 0.1 ^b^
Methyl octanoate	1111	2 × 10^−1^ [36]	Intense citrus	2.2 ± 0.0	nd	3.1 ± 0.1
Ethyl octanoate	1429	2 × 10^−3^ [5]	Pineapple, pear, floral	4383.9 ± 77.5 ^c^	6263.5 ± 139.8 ^a^	5741.8 ± 44.2 ^b^
Isoamyl hexanoate	2044	NA	NA	tr	tr	tr
Ethyl decanoate	1637	2 × 10^−1^ [5]	Fruity, fatty, pleasant	861.8 ± 2.8 ^b^	1148.7 ± 45.6 ^a^	1200.5 ± 77.0 ^a^
Diethyl succinate	1682	200 [41]	Light fruity	766.2 ± 33.4 ^c^	1456.9 ± 89.6 ^a^	1123.0 ± 100.5 ^b^
Ethyl dodecanoate	1848	1.5 [36]	Flowery, fruity	698.5 ± 2.9 ^c^	1672.4 ± 59.9 ^b^	3375.1 ± 77.7 ^a^
Subtotal (μg/L)				129003.8	187738.1	207985.1
Proportion (%)				56.1	54.6	56.6
Acids						
Isobutyric acid	1607	200 [5]	Fatty	nd	24.7 ± 1.3	nd
Hexanoic acid	1855	3 [5]	Cheese, rancid, fatty	1737.4±54.2 ^b^	4113.2 ± 231.0 ^a^	3961.0 ± 37.1 ^a^
Heptanoic acid	1990	3 [42]	Fatty, dry	tr	nd	nd
Octanoic acid	2075	5 × 10^−1^ [5]	Rancid, harsh, cheese, fatty acid	1834.4 ± 137.8 ^b^	4094.4 ± 97.8 ^a^	3875.1 ± 233.4 ^a^
Decanoic acid	2292	15 [5]	Fatty, unpleasant	501.0 ± 100.8 ^b^	1483.5 ± 30.8 ^a^	1404.1 ± 74.5 ^a^
Subtotal (μg/L)				4072.8	9715.8	9240.2
Proportion (%)				1.8	2.8	2.5
Aldehydes and ketones						
Nonanal	1394	1 × 10^−3^ [43]	Green, slightly pungent	tr	tr	nd
Benzaldehyde	1534	2 [35]	Almond	42.0 ± 2.5 ^a^	11.7 ± 0.1 ^b^	11.4 ± 0.4 ^b^
Benzylethylaldehyde	1782	NA	NA	55.0±3.7	nd	nd
Geranylacetone	1864	6 × 10^−2^ [36]	Floral	tr	nd	5.5 ± 0.2
Acetoin	1284	150 [5]	Flowery, wet	2343.2 ± 50.7 ^c^	4009.7 ± 138.9 ^a^	3064.3 ± 67.7 ^b^
Subtotal (μg/L)				2440.2	4021.4	3081.2
Proportion (%)				1.1	1.2	0.8
Others						
Phenol	2006	NA	NA	0.6 ± 0.0 ^c^	2.4±0.1 ^a^	1.8 ± 0.2 ^b^
Subtotal (μg/L)				0.6	2.4	1.8
Proportion (%)				< 0.1	< 0.1	<0 .1
Total (μg/L)				230136.7	343594.0	367186.9

The data were mean values ± standard deviation of triplicate samples. Different letters within a row for the same aromatic compound indicated significant differences among three terrain wines by Tukey’s test (*p* < 0.05). Retention indices (RI) were on the poly(ethylene glycol) (PEG) column. RI, compounds were identified by a comparison to the pure standard. NA, not apply. nd, not detected. tr, trace.

**Table 3:** OAVs of the aroma compounds in Cabernet Franc wines.

Compounds	Threshold (mg/L)	Sensory properties	F-Land	LS-Land	HS-Land
Ethyl hexanoate	5 × 10^−3^ [5]	Fruity, anise	10218.1	12772.1	12925.5
Ethyl octanoate	2 × 10^−3^ [5]	Pineapple, pear, floral	2192.0	3131.8	2870.9
Isoamyl acetate	3 × 10^−2^ [5]	Banana	74.6	52.3	68.4
Ethyl acetate	7.5 [5]	Fruity, sweet	8.6	14.3	16.5
Ethyl butanoate	2 × 10^−2^ [5]	Sour fruit, fruity	nd	nd	13.3
Octanoic acid	5 × 10^−1^ [5]	Rancid, harch, cheese, fatty acid	3.7	8.2	7.8
Ethyl decanoate	2 × 10^−1^ [5]	Fruity, fatty, pleasant	4.3	5.7	6.0
3-(Methylthio)-1--propanol	5 × 10^−1^ [5]	Boiled potato, rubber	4.1	5.6	5.8
Ethyl dodecanoate	1.5 [36]	Flowery, fruity	0.5	1.1	2.3
2-Phenylethanol	10 [5]	Flowery, pollen, perfume	1.1	3.1	3.1
Isoamyl alcohol	30 [5]	Cheese	1.7	1.8	2.2
(*Z*)-3-Hexen-1-ol	4 × 10^−1^ [5]	Green	0.1	1.5	0.3
Hexanoic acid	3 [5]	Cheese, rancid, fatty	0.6	1.4	1.3

nd, not detected.

**Table 4:** Characteristics of three experimental localities.

Locality	North latitude	East longitude	Altitude (m)	Aspect	Slope (%)
F-Land	35°59′59″	110°46′48″	1201	NA	NA
LS-Land	36°01′38″	110°49′00″	1323	SN	6.2
HS-Land	36°02′41″	110°48′43″	1381	SN	13.2

NA, not applicable. SN, South-north, it represents the row aspect of the experimental vineyard.
